# Electrophysiological Correlation Patterns of Resting State Networks in Single Subjects: A Combined EEG–fMRI Study

**DOI:** 10.1007/s10548-012-0235-0

**Published:** 2012-06-30

**Authors:** Matthias C. Meyer, Erik S. B. van Oort, Markus Barth

**Affiliations:** 1 Radboud University Nijmegen, Donders Institute for Brain, Cognition and Behaviour, Nijmegen, The Netherlands; 2 MIRA Institute for Biomedical Technology and Technical Medicine, University of Twente, Enschede, The Netherlands; 3 Erwin L. Hahn Institute for Magnetic Resonance Imaging, University Duisburg-Essen, Essen, Germany

**Keywords:** Combined EEG–fMRI, Resting state networks, Single-subject, ICA, Dual regression, ECP

## Abstract

With combined EEG–fMRI a powerful combination of methods was developed in the last decade that seems promising for answering fundamental neuroscientific questions by measuring functional processes of the human brain simultaneously with two complementary modalities. Recently, resting state networks (RSNs), representing brain regions of coherent BOLD fluctuations, raised major interest in the neuroscience community. Since RSNs are reliably found across subjects and reflect task related networks, changes in their characteristics might give insight to neuronal changes or damage, promising a broad range of scientific and clinical applications. The question of how RSNs are linked to electrophysiological signal characteristics becomes relevant in this context. In this combined EEG–fMRI study we investigated the relationship of RSNs and their correlated electrophysiological signals [electrophysiological correlation patterns (ECPs)] using a long (34 min) resting state scan per subject. This allowed us to study ECPs on group as well as on single subject level, and to examine the temporal stability of ECPs within each subject. We found that the correlation patterns obtained on group level show a large inter-subject variability. During the long scan the ECPs within a subject show temporal fluctuations, which we interpret as a result of the complex temporal dynamic of the RSNs.

**Electronic supplementary material:**

The online version of this article (doi:10.1007/s10548-012-0235-0) contains supplementary material, which is available to authorized users.

## Introduction

During the last decade, the combination of electroencephalography and functional magnetic resonance imaging (EEG–fMRI) in humans became a prevalent tool in neuroscience and related research fields. Using methods based on triggered average subtraction of MR related cardiac and gradient artifacts in the EEG, as first formulated by Allen et al. (1998) and Allen et al. (2000), the electrophysiological signal could be filtered sufficiently to enable the examination of neuroscience questions, making combined EEG–fMRI a potentially powerful tool for noninvasive investigation of human brain function. And indeed, studies examining global EEG characteristics and corresponding BOLD signals reported significant correlations between the EEG theta, alpha or beta band power and BOLD signal fluctuation in specific brain regions (Goldman et al. 2002; Laufs et al. 2003a, b, 2006; Moosmann et al. 2003; Feige et al. 2005; Goncalves et al. 2006; Scheeringa et al. 2008). These studies used EEG derived regressors for different frequency bands to correlate with the fMRI time course. Their findings however are rather mixed and inconclusive, due to the resulting inconsistent BOLD maps. This lead to the assumption that several frequency bands might be involved in distinct functional networks (Laufs et al. 2006; Mantini et al. 2007).

Around the same time, BOLD resting state networks (RSNs), which represent coherently fluctuating brain regions observed in the resting human brain, became a topic of major interest (Biswal et al. 1995; Lowe et al. 2000; Cordes et al. 2001; Greicius et al. 2003; Fox et al. 2005; Damoiseaux et al. 2006; De Luca et al. 2006; Smith et al. 2009; Laird et al. 2011). Using Independent Component Analysis (ICA), Damoiseaux et al. (2006) showed that these RSNs are consistent across subjects. Recently, it was demonstrated by Smith et al. (2009), that ten of these RSNs reflect various known task related networks (TRNs) obtained from a large database of functional studies. However, the question of the direct relation between both modalities in the resting brain is currently intensely investigated (Mantini et al. 2007; Goncalves et al. 2008; Britz et al. 2010; Laufs 2010; Schölvinck et al. 2010). Mantini et al. (2007) investigated the correlation of various EEG frequency bands with these RSNs using EEG–fMRI and found evidence for specific group level EEG band power correlation profiles for six RSNs. However, studies relating the alpha rhythm on subject level to coherently fluctuating BOLD signals during resting state (RS) (Goncalves et al. 2006,  2008) observed considerable inter-subject variability in the correlation between both modalities. Given the low inter-subject variability in the BOLD characteristics, they concluded that the observed inter-subject variability arises from the individual variation in the EEG.

In this combined EEG–fMRI study we investigated the relation of the electrophysiological signal to ICA derived RSNs which resemble TRNs (Smith et al. 2009). The link between RSNs as found by ICA and the electrophysiological signal was obtained by correlating EEG frequency power of four common, low frequency bands (δ: (2–4) Hz, θ: (4–7) Hz, α: (8–12) Hz, β: (12–30) Hz) to a specific RSN, resulting in electrophysiological correlation patterns (ECPs). Using a long, 34 min resting state scan per subject we hypothesized that we could investigate the ECPs not only on group, but also on a single subject level, and, furthermore, would be able to assess the temporal stability of these ECPs within a subject.

## Data and Methods

### Data Acquisition

#### (f)MRI

For this study, 16 healthy subjects, 12 female and four male with a mean age of 22 (range 19–41) were scanned. The experiment was approved by the local ethical committee. For each subject, 34 min of resting state data were acquired using a 3T Magnetom TIM Trio system (Siemens Healthcare, Erlangen, Germany) with the product 32 channel head coil and a multi echo EPI sequence (Poser et al. 2006) (acquisition parameters: 1030 Vol., TR = 2000 ms, five echoes: TE = 6.9, 16.2, 25, 35 and 44 ms, flip angle 80°, 39 slices, 3.5 mm isotropic resolution, GRAPPA factor 3, 6/8 partial Fourier). A T1 structural scan was obtained to register the functional data to Montreal neurological institute (MNI) space using an MPRAGE protocol (acquisition parameters: voxel size 1.0 × 1.0 × 1.0 mm^3^, matrix size 256 × 256, 192 slices, TR = 2300 ms, TE = 3.03 ms, T1 = 1100 ms, flip angle = 8°).

#### EEG

EEG data were recorded simultaneously with a 32 channel cap (ANT WaveGuard MRI), using a BrainAmp MR plus amplifier and BrainVision Recorder (sample frequency = 5000 Hz, resolution = 0.5 μV, operating range = ± 16.384 mV, hardware high pass filter 0.1 Hz and low pass filter at 250 Hz) (BrainVision, Gilching, Germany). The last 4 subjects were recorded with a 64 channel cap (BrainVision). To stay comparable with the previous experiments in this study only the same 30 channels (10–20 system) were used in the analysis. The MR gradient clock and the EEG amplifier clock were synchronized for optimal gradient artifact correction.

The subjects were asked to relax, keep their eyes open, stay awake and not think of anything specific. The room was darkened during the scan and an infrared eye tracker was used to control that the subject would not fall asleep. All subjects managed to stay awake for the complete duration of the experiment.

12 of the 16 datasets were used for analysis. Four data sets were excluded due to: incomplete recording of the EEG due to technical problems; saturation of the EEG channels caused by large gradient artifacts; residual gradient artifacts after correction caused by syncing problems between MR scanner and EEG amplifier; and abnormal brain anatomy, respectively.

This combined EEG–fMRI study was part of a larger RS study, including 77 subjects, for which an additional diffusion weighted scan was recorded (1 h) and a behavioral questionnaire was filled in during a break after the RS scan.

### Preprocessing

#### (f)MRI

RS–fMRI preprocessing was performed using functions from the SPM5 software package (Welcome Department of Imaging Neuroscience, University College London, UK). The used multi-echo sequence acquired one volume for each of the five echoes at every time point. To allow the use of standard fMRI preprocessing tools, these five echoes were combined using the procedure described in Poser et al. (2006). In short, the first 30 time points were used to calculate the temporal signal-to-noise ratio for every voxel, needed to obtain the echo specific weighting factors for the echo combination. SPM5 motion correction was applied to the first echoes and the calculated corrections were applied to every subsequent echo of the same volume. It can safely be assumed that the subjects do not move between echoes, because of the short duration of the multi-echo train of about 50 ms. The first six volumes were discarded to allow the system to reach a steady state. The functional data from every subject were spatially smoothed with a 5 mm Gaussian kernel and transformed to MNI space using FSL’s Feat (FSL 4.1, www.fmrib.ox.ac.uk/fsl/).

#### EEG

MR related artifacts in the EEG signal were removed using Analyzer 2 (BrainVision). Trigger based average subtraction (Allen et al. 2000) was used to correct for gradient artifacts and subsequently the data were downsampled to 500 Hz. A Butterworth zero phase filter, 48 dB/oct with a low cutoff at 0.8 Hz, to remove slow fluctuations from respiration, and a high cutoff of 50 Hz was applied. Additionally a notch filter at 50 Hz was applied to remove residual mains frequency noise.

Cardiac related MR artifacts were removed using the Adaptive Average Subtraction (AAS) method. This was done in three steps: First, markers were obtained by the algorithm implemented in Analyzer2 that detects MR related cardiac artifacts, using a channel with cardiac artifacts that remained quite constant in amplitude and were well visible. Next, all markers were checked manually by visual inspection and, finally, the AAS algorithm was applied. ICA was used to remove eye blinks. The EEG data were re-referenced to common average.

### Analysis

#### (f)MRI

**Fig. 1:**
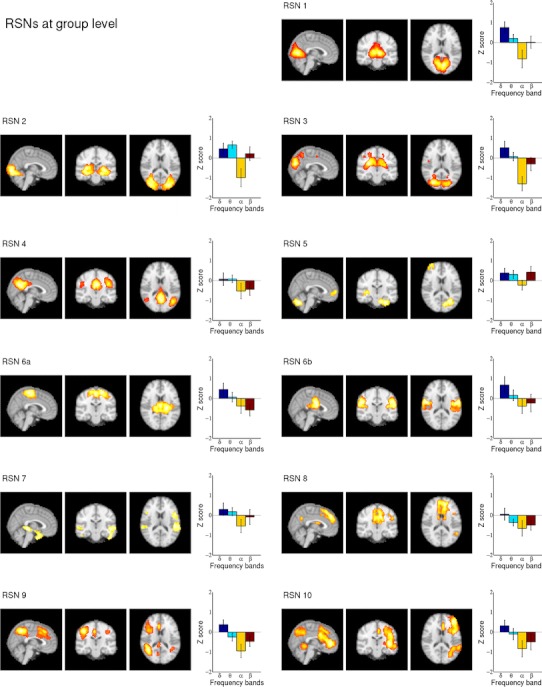
All 11 group fMRI RSNs as maximum intensity projection on the central slices and their group ECPs, representing the average Z scores (12 subjects) for the four EEG frequency bands. Only clusters larger than 15 voxels were plotted. The large standard errors indicate the large variability of the subject-specific ECPs

The normalized fMRI data were concatenated temporally and group ICA was performed using FSL’s Melodic 3.1 to obtain 30 group independent components (ICs). The number of components was chosen for optimal noise separation and integrity of the RSNs. A higher number could cause the RSNs to split up into sub networks (Smith et al. 2009; Kiviniemi et al. 2009). These 30 group IC maps were used in a dual regression approach (Filippini et al. 2009) to obtain subject specific IC maps. These maps were thresholded (Z ≥ 6) and used as masks to obtain an average BOLD time course of the IC on subject level. We find all RSNs reported by Smith et al. (2009) and, furthermore, ten components that show also characteristics of RSNs regarding their functional maps and their frequency spectra. The remaining nine components contain white matter, movement artefacts, or breathing related artefacts, a.s.o. For further analysis we selected eleven RSNs (see Fig. 1), which resemble those described by Smith et al. (2009) depicting TRNs. The sensorimotor component RSN 6 reported by Smith et al. (2009) split up into two components, termed RSN 6a and RSN 6b.

#### EEG

Corresponding to the TR used in the fMRI acquisition the EEG signal was split into 2 s segments based on the MR volume trigger. A Fourier transform was applied to each channel in the segment and the frequency power spectrum of all channels was averaged. The power spectra were split into four frequency bands: δ: 2–4 Hz, θ: 4–7 Hz, α: 8–12 Hz, β: 12–30 Hz and the power spectrum within each band was integrated, resulting in one power time course for each EEG band. The four power time courses were convolved with the standard SPM5 hemodynamic response function (HRF) and correlated with the BOLD time course of each RSN using partial correlation to account for common variance (De Munck et al. 2009), resulting in a subject specific correlation value per frequency band. Z-values were calculated on basis of these correlation values, using the mean of all correlation values as global mean. Group level results were obtained by averaging Z-values over subjects per frequency band and the variation was calculated as the standard error of the mean.

To estimate the influence of variations of the HRF on the ECPs we varied the temporal shifts of the BOLD HRF by a large, but physiologically plausible amount (Aguirre et al. 1998), and shifted the signals by two seconds in both directions relative to each other before correlating.

To assess the temporal behavior of the ECPs within a single subject the dataset was split into five equal parts (>6 min) and the same procedure as described above was performed on each of the parts.

Correlation analysis was performed and figures were made using MATLAB (R2010b, The MathWorks, Natick, Massachusetts, USA).

## Results

A list of the eleven RSNs, which correspond to those found by Smith et al. (2009) and which we used in the correlation analysis, is given in Table 1. Figure 1 shows these group RSNs as maximum intensity projection on the central slices, together with their group ECPs, representing the average Z scores of the 12 subjects. The group ECPs of the 11 RSNs are not significant and show a large standard error of the mean, indicating a large variability of subject specific ECPs. The three visual components (RSN 1–3) however show a relatively high negative correlation with the alpha band.

**Table 1:** Lists the RSNs found using ICA and their classification according to Smith et al. (2009)

RSN 1	Medial visual component
RSN 2	Occipital pole component
RSN 3	Lateral visual component
RSN 4	Default mode network (DMN)
RSN 5	Cerebellum (here combined with a lateralized anterior component)
RSN 6a	Sensory motor component
RSN 6b	Lower sensory motor component
RSN 7	Auditory system
RSN 8	Executive control—medial frontal network
RSN 9	Frontoparietal lateralized network (left)
RSN 10	Frontoparietal lateralized network (right)

**Fig. 2:**
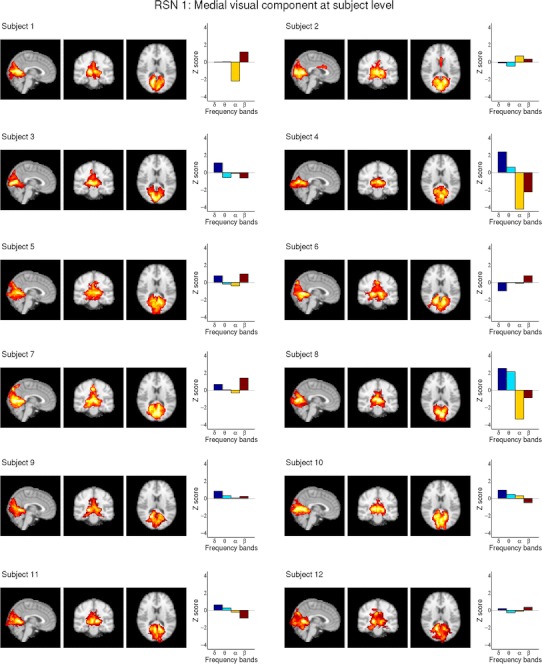
Depicts RSN 1 (medial visual component) after dual regression on single subject level as maximum intensity projection on the central slices and the subject specific ECPs for all 12 subjects, showing the high inter-subject variability of the ECPs but also significant negative alpha correlation in subject 4 and subject 8. For visualization purposes a cluster threshold of 100 voxels and a minimum intensity threshold of 25 % was used

**Fig. 3:**
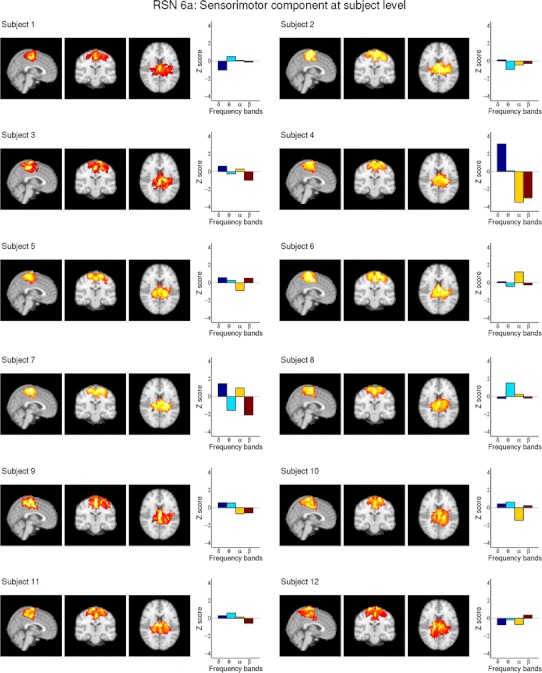
Depicts RSN6a (sensorimotor component) after dual regression on single subject level as maximum intensity projection on the central slices and the subject specific ECPs for all 12 subjects, showing the high inter-subject variability of the ECPs. Subject 4 shows significant positive delta correlation and negative alpha and beta correlation. For visualization purposes a cluster threshold of 100 voxels and a minimum intensity threshold of 25 % was used

Figures 2 and 3 show two selected RSNs (RSN 1, medial visual component and RSN 6a, sensorimotor component) and their ECPs for all 12 subjects, which clearly shows the subject specific characteristics of the individual ECPs. At Z > 2.58 (corresponding to a *p* value <0.05 corrected for multiple comparisons) four of the twelve subjects showed significant correlation with at least one RSN. Subject 1 shows significant negative alpha correlation with RSN 3 and RSN 4. Subject 4 exhibits significant correlations in 10 of the 11 RSNs, which consistently show negative alpha and beta correlations as well as positive delta correlations. Subject 5 shows significant negative alpha correlation in RSN 11. Subject 8 shows negative alpha correlation as well as positive delta correlation in four RSNs. See Table 2 for a detailed list of all significant correlations. Three of these four subjects show negative alpha correlation with visual RSNs (see also supplementary Figs. S1 and S2).

**Table 2:** lists all significant ECPs (Z scores) sorted by frequency band, subjects and RSNs

	RSN1	RSN2	RSN3	RSN4	RSN6a	RSN6b	RSN7	RSN8	RSN9	RSN10
Delta										
Subject4				2.81	3.15	2.69				2.63
Subject8						3.69				
Alpha										
Subject1		−3.04		−3.01						
Subject4	−4.21	−4.22	−3.85	−3.42	−3.47	−3.38	−3.68	−3.98	−3.58	−3.83
Subject5										−3.04
Subject8	−3.33	−2.93	−3.23							
Beta										
Subject4			−2.75	−2.77	−2.98	−3.84	−2.83			−3.86

The sensorimotor component reported by Smith et al. (2009) was split into two sub components RSN 6a (Fig. 3) and RSN 6b (supplementary Fig. S3). Subject 4 shows very similar ECPs for both RSNs with significant correlation in the delta, alpha and beta band. The ECPs of subject 8 however differ a lot between RSN 6a and 6b, showing significant correlation with the delta band in RSN 6b and no significant correlation in RSN 6a.

To assess the influence of the variation of the HRF on the ECPs by subject specific differences we varied the temporal shifts of the BOLD HRF, i.e. shifting the time courses two seconds relative to each other before correlating. This slightly changed the correlation values at the group level. However the shapes of the ECPs were quite stable. On subject level the changes of the specific ECPs due to shifting were larger and in a few non significant instances even lead to a sign switch (negative correlation instead of positive in a frequency band). A reduction in inter-subject variability of the specific ECPs across the different shifts was not observed. Furthermore, the same four subjects showed significant correlation across all shifts, with only small changes of the ECPs.

**Fig. 4:**
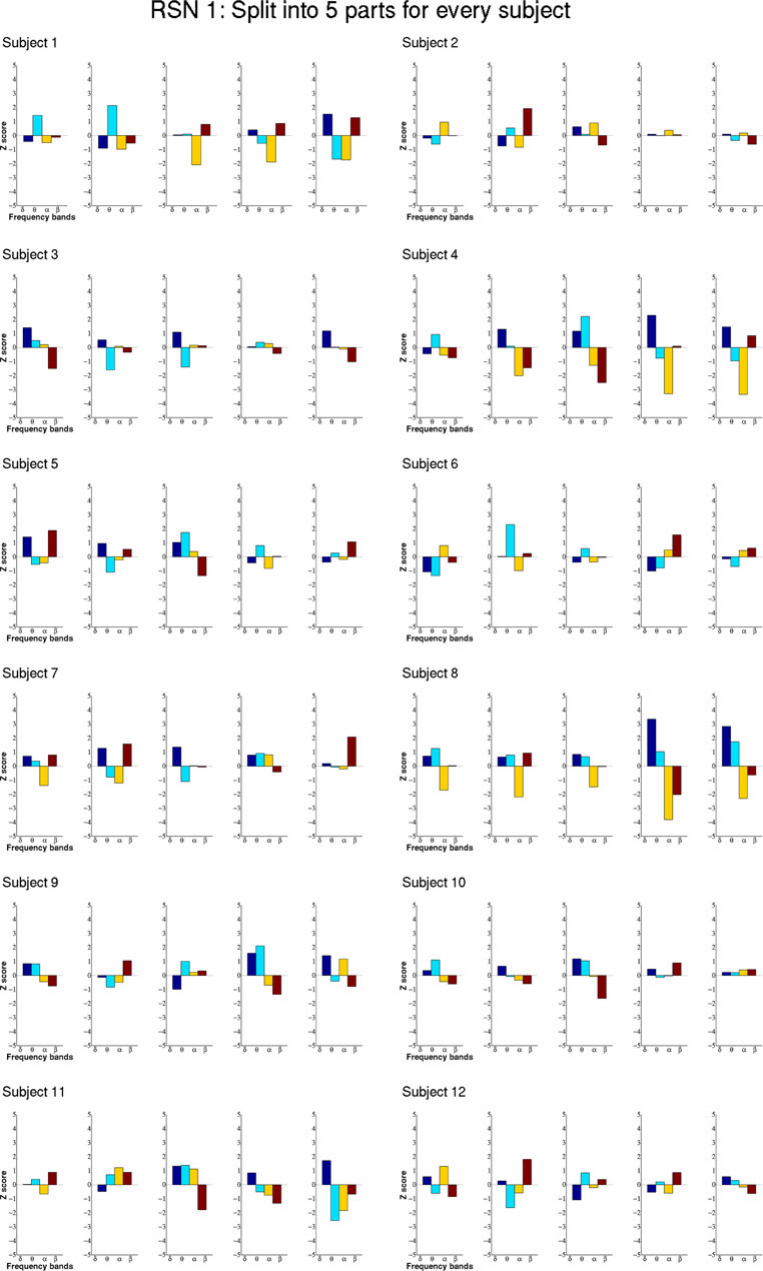
Shows the ECPs of RSN 1 (medial visual component) for all five parts of the split datasets for all 12 subjects. The ECPs show higher Z scores at these shorter time intervals and the patterns change over time

**Fig. 5:**
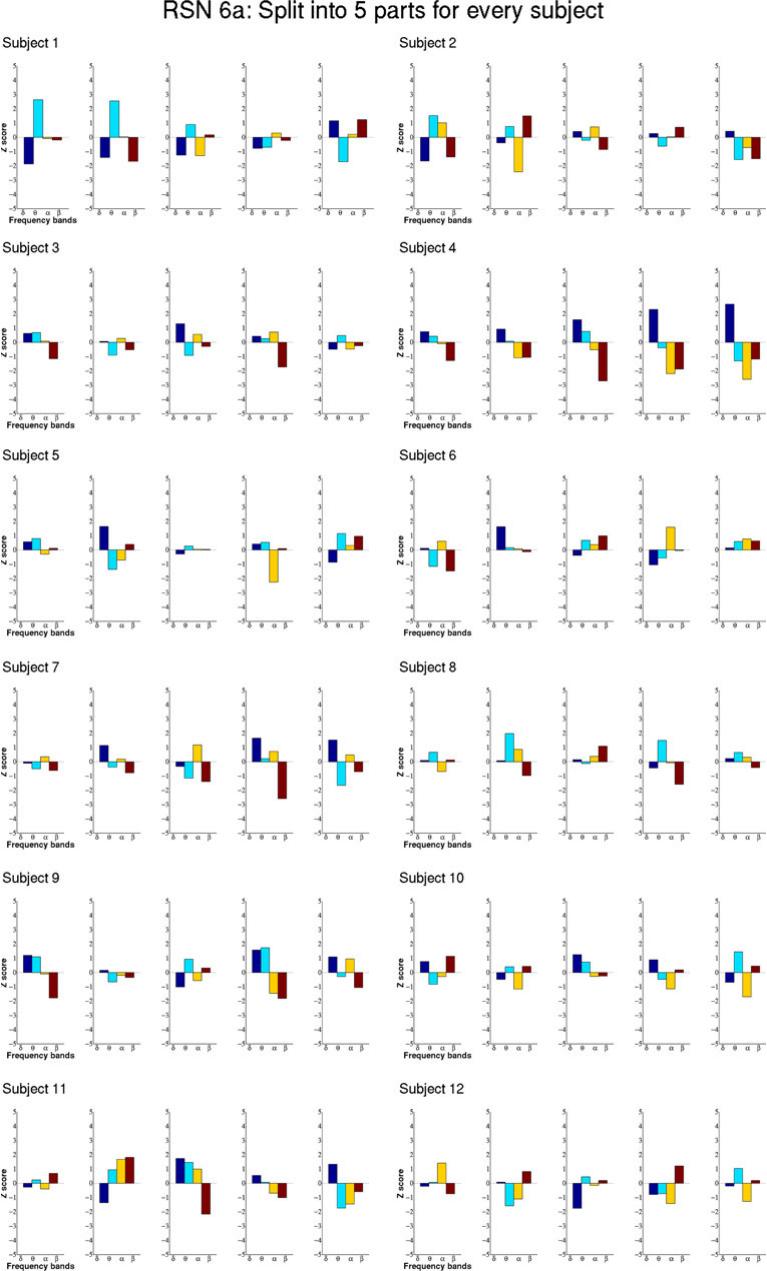
Shows the ECPs of RSN 6a (sensorimotor component) for all five parts of the split datasets for all 12 subjects. The ECPs show higher Z scores at these shorter time intervals and the patterns change over time

Due to the long resting state acquisition, we were able to also investigate the temporal variation of ECPs by splitting the datasets into five parts where each is still of sufficient length. Figs. 4 and 5 show the five ECPs for every part of the datasets of two exemplary RSNs, again RSN 1 and 6a, for all subjects. There is a certain resemblance of the ECPs within a subject, however, also a clear temporal variation. Furthermore, one can see significant changes in the ECPs. For example, subject 4 seems to have a state shift in the middle of the acquisition. Compared to the ECPs of the complete session most of the subjects show higher correlation values at these shorter intervals.

## Discussion

Our findings show that BOLD RSNs found with ICA and dual regression are very similar in all subjects, but the corresponding ECPs show large variations between subjects leading to a non-significant correlation on group level. On subject level we found significant correlation in four of the twelve subjects with consistent negative alpha correlation with the three visual RSNs, which is in good agreement with the common finding in previous studies (Goldman et al. 2002; Moosmann et al. 2003; Laufs et al. 2006; Goncalves et al. 2006). Apart from this, these studies used EEG derived regressors, which lead to large variation of the BOLD maps. In contrast to these studies we employed a well established explorative and automated approach to derive stable BOLD RSNs across subjects whose time course was correlated with the EEG. Thus, the observed inter-subject variability very likely results from the EEG, which would explain the mixed results found in the former studies.

Our findings are consistent with the finding of Goncalves et al. (2008) where a large inter-subject variability of the correlation between EEG and fMRI was reported for the alpha frequency band. Furthermore, they interpreted the results such that this variability arises from the EEG, since the BOLD activation clusters were stable over subjects, which is consistent with our observations. Their and our findings are, however, in contrast to a study by Mantini et al. (2007) who reported significant EEG correlation patterns for six BOLD RSNs on group level with a relatively low inter-subject variation. This could be due to differences in the experimental setup (length of the resting state scan, eyes open vs. eyes closed), but also due to differences in the analysis. Mantini et al. (2007) used single subject ICA in combination with a clustering algorithm to obtain group maps.

In our attempt to pin down possible sources of this inter-subject variability we performed several analysis steps. At first we investigated the influence on the ECPs by subject specific differences of the HRF, which links electrophysiological activity and BOLD (Friston et al. 1998a, b; Logothetis et al. 2001; Stephan et al. 2007). To probe the influence of HRF variations we varied the temporal shifts of the HRF, which slightly changed the correlation values on group level, however the shapes of the ECPs were quite stable. A larger influence was observed on single subject level. Obviously, the inter-individual variation in HRFs influences the ECPs and is a very important topic to address in terms to understand in detail the relation between both modalities (Schippers et al. 2011; Schölvinck et al. 2010; Stephan et al. 2007), but from our results it seems that inter-subject differences in the HRF are not the main reason for the observed inter-subject variability in the ECPs. To investigate the influence of electrode selection on the variability of ECPs we selected electrodes that are spatially close to the related RSNs (frontal and occipital). This did not improve the specificity of the ECPs compared to averaging over all channels, as the ECPs showed high similarities between an anterior and posterior selection of electrodes due to volume conduction. A simple manual selection of electrodes did not improve the specificity of the ECPs and did not lead to a reduced inter-subject variability.

Finally we examined the temporal stability of the ECPs within individual subjects by splitting the individual datasets into five equal segments. The ECPs for the different RSNs were calculated for each segment, showing large fluctuations over the entire scan. On the other hand, the observed temporal changes of the ECPs do not seem to be arbitrary. In essence, some individual characteristics are kept and exhibit mostly smooth temporal changes. However, it is clear that intra-individual temporal changes of the ECPs lead to a higher inter-individual variability. Most probably these temporal changes of the ECPs can be explained by the temporal dynamics of the RSNs as observed in some studies (e.g. Damoiseaux et al. 2006). Thus, correlating the power timelines of the different EEG frequency bands with one specific RSN time course will lead to low and unstable correlation values because they consist of the superposition of the electrophysiological signals coming from all RSNs, which cannot be disentangled by a global EEG power time course. This might also explain why more significant correlation could be found in shorter intervals. An alternative explanation for our observations would be that the different RSNs do not have a specific ECP, but that different states of one RSN lead to different ECPs. Our observation of the temporally changing ECPs is in good agreement with findings by Schölvinck et al. (2010), who reported changes of correlation patterns over time in a combined EEG–fMRI resting state experiment with macaque monkeys. They found alertness related fluctuations of the correlation between both modalities in the gamma frequency range in two of the three participating monkeys whereby a lower correlation was reported during eyes open RS. This might be one possible explanation for the low correlations found in our eyes-open resting state study. They also report a huge variability between the animals in the correlation between EEG and BOLD for the lower frequency range. An overview of combined EEG–fMRI, including animal studies, can be found in the review of Leopold and Maier (2011).

In future studies, to more specifically investigate the relation between RSNs and EEG, one would need a direct handle to link specific EEG components to the different RSNs. To use temporal ICA for the analysis of resting state EEG equivalent to those studies using a task design (e.g. Eichele et al. 2008) is not possible since clear characteristic temporal patterns are missing. A more promising solution might be the separation of EEG on the basis of the fMRI RSNs which are stable across subjects. We are of the opinion that by using a realistic head model (Acar and Makeig 2010; Bojak et al. 2011; Hallez et al. 2007) one could in principle directly model the link between fMRI RSNs and their related EEG for more specific investigations of the relation between both modalities in a resting state.

In summary, we found reproducible RSNs across subjects and significant correlations with EEG in four of the twelve subjects, three of them showed negative alpha correlation with visual RSNs which is in good agreement with previous findings. However, we also observed large inter-subject variability in the ECPs. Besides a clear inter-individual difference in EEG patterns, it seems that the found temporal variability of the ECPs within a subject explains a large part of the observed inter-subject variability in the ECPs, i.e. the correlation between EEG frequency power and BOLD RSNs. In addition, the unstable ECPs over time between both modalities might be inherent to eyes open RS. It seems that there is only a minor contribution from the variation of the individual HRF to the inter-subject variability of the ECPs.

## Electronic supplementary material

Below is the link to the electronic supplementary material.

Supplementary material 1 (TIFF 3196 kb)

Supplementary material 2 (TIFF 3253 kb)

Supplementary material 3 (TIFF 3184 kb)

## Abbreviations

EEG: Electroencephalography
(f)MRI: (functional) Magnetic resonance imaging
RS: Resting state
RSN: Resting state network
BOLD: Blood oxygen level dependent
ECP: Electrophysiological correlation patterns
IC: Independent component
ICA: Independent component analysis
TRN: Task related network
EPI: Echo planar imaging
GRAPPA: Generalized autocalibrating partially parallel acquisition
MPRAGE: Magnetization prepared rapid gradient echo
TR: Repetition time
TE: Echo time
MNI: Montreal neurological institute
SPM: Statistical parametric mapping
FSL: FMRIB Software Library
AAS: Adaptive average subtraction
HRF: Hemodynamic response function
DMN: Default mode network

## References

[CR1] Acar Z, Makeig S (2010). Neuroelectromagnetic forward head modeling toolbox. J Neurosci Methods.

[CR2] Aguirre GK, Zarahn E, D’esposito M (1998). The variability of human. BOLD hemodynamic responses. NeuroImage.

[CR3] Allen PJ, Polizzi G, Krakow K, Fish DR, Lemieux L (1998). Identification of EEG events in the MR scanner: the problem of pulse artifact and a method for its subtraction. NeuroImage.

[CR4] Allen PJ, Josephs O, Turner R (2000). A method for removing imaging artifact from continuous EEG recorded during functional MRI. NeuroImage.

[CR5] Biswal B, Yetkin FZ, Haughton VM, Hyde JS (1995). Functional connectivity in the motor cortex of resting human brain using echo-planar MRI. Magn Reson Med.

[CR6] Bojak I, Oostendorp TF, Reid AT, Kötter R (2011). Towards a model-based integration of co-registered electroencephalography/functional magnetic resonance imaging data with realistic neural population meshes. Philos Trans A Math Phys Eng Sci..

[CR7] Britz J, Van De Ville D, Michel CM (2010). BOLD correlates of EEG topography reveal rapid resting-state network dynamics. NeuroImage..

[CR8] Cordes D, Haughton VM, Arfanakis K, Carew JD, Turski PA, Moritz CH, Quigley MA, Meyerand ME (2001). Frequencies contributing to functional connectivity in the cerebral cortex in “resting-state” data. AJNR.

[CR9] Damoiseaux JS, Rombouts SA, Barkhof F, Scheltens P, Stam CJ, Smith SM, Beckmann CF (2006). Consistent resting-state networks across healthy subjects. Proc Natl Acad Sci USA.

[CR10] De Luca M, Beckmann CF, De Stefano N, Matthews PM, Smith SM (2006). fMRI resting state networks define distinct modes of long-distance interactions in the human brain. NeuroImage.

[CR11] De Munck JC, Gonçalves SI, Mammoliti R, Heethaar RM, Lopes da Silva FH (2009). Interactions between different EEG frequency bands and their effect on alpha-fMRI correlations. Neuroimage..

[CR12] Eichele T, Calhoun VD, Moosmann M, Specht K, Jongsma ML, Quiroga RQ, Nordby H, Hugdahl K (2008). Unmixing concurrent EEG–fMRI with parallel independent component analysis. Int J Psychophysiol.

[CR13] Feige B, Scheffler K, Esposito F, Di Salle F, Hennig J, Seifritz E (2005). Cortical and subcortical correlates of electroencephalographic alpha rhythm modulation. J Neurophysiol.

[CR14] Filippini N, Macintosh BJ, Hough MG, Goodwin GM, Frisoni GB, Smith SM, Matthews PM (2009). Distinct patterns of brain activity in young carriers of the APOE-epsilon4 allele. Proc Natl Acad Sci USA.

[CR15] Fox MD, Snyder AZ, Vincent JL, Corbetta M, Van Essen DC, Raichle ME (2005). The human brain is intrinsically organized into dynamic, anticorrelated functional networks. Proc Natl Acad Sci USA.

[CR16] Friston KJ, Josephs O, Rees G, Turner R (1998). Nonlinear event-related responses in fMRI. Magn Reson Med.

[CR17] Friston KJ, Fletcher P, Josephs O, Holmes A (1998). Event-Related fMRI: Characterizing Differential Responses. NeuroImage.

[CR18] Goldman RI, Stern JM, Engel J, Cohen MS (2002). Simultaneous EEG and fMRI of the alpha rhythm. NeuroReport.

[CR19] Goncalves SI, de Munck JC, Pouwels PJ, Schoonhoven R, Kuijer JP, Maurits NM, Hoogduin JM (2006). Correlating the alpha rhythm to BOLD using simultaneous EEG/fMRI: inter-subject variability. NeuroImage.

[CR20] Goncalves SI, Bijma F, Pouwels PJ, Jonker M, Kuijer JP, Heethaar RM, Lopes da Silva FH, de Munck JC (2008). A data and model-driven approach to explore inter-subject variability of resting-state brain activity using EEG–fMRI. IEEE Journal of Selected topics in signal processing.

[CR21] Greicius MD, Krasnow B, Reiss AL, Menon V (2003). Functional connectivity in the resting brain: a network analysis of the default mode hypothesis. Proc Natl Acad Sci USA.

[CR22] Hallez H, Vanrumste B, Grech R, Muscat J, De Clercq W, Vergult A, D’asseler Y (2007). Review on solving the forward problem in EEG source analysis. J Neuroeng Rehabil.

[CR23] Kiviniemi V, Starck T, Remes J, Long X, Nikkinen J, Haapea M, Veijola J (2009). Functional segmentation of the brain cortex using high model order group PICA. Hum Brain Mapp.

[CR24] Laird AR, Fox PM, Eickhoff SB, Turner JA, Ray KL, McKay DR, Glahn DC (2011). Behavioral Interpretations of Intrinsic Connectivity Networks. J Cogn Neurosci.

[CR25] Laufs H (2010). Multimodal analysis of resting state cortical activity: what does EEG add to our knowledge of resting state BOLD networks?. NeuroImage.

[CR26] Laufs H, Kleinschmidt A, Beyerle A, Eger E, Salek-Haddadi A, Preibisch C, Krakow K (2003). EEG-correlated fMRI of human alpha activity. NeuroImage.

[CR27] Laufs H, Krakow K, Sterzer P, Eger E, Beyerle A, Salek-Haddadi A, Kleinschmidt A (2003). Electroencephalographic signatures of attentional and cognitive default modes in spontaneous brain activity fluctuations at rest. Proc Natl Acad Sci USA.

[CR28] Laufs H, Holt JL, Elfont R, Krams M, Paul JS, Krakow K, Kleinschmidt A (2006). Where the BOLD signal goes when alpha EEG leaves. NeuroImage.

[CR29] Leopold DA, Maier A (2011) Ongoing physiological processes in the cerebral cortex, NeuroImage [Epub ahead of print] doi: 10.1016/j.neuroimage.2011.10.059 10.1016/j.neuroimage.2011.10.059 PMC3288739 22040739

[CR30] Logothetis NK, Pauls J, Augath M, Trinath T, Oeltermann A (2001). Neurophysiological investigation of the basis of the fMRI signal. Nature.

[CR31] Lowe MJ, Dzemidzic M, Lurito JT, Mathews VP, Phillips MD (2000). Correlations in low-frequency BOLD fluctuations reflect cortico-cortical connections. NeuroImage.

[CR32] Mantini D, Perrucci MG, Del Gratta C, Romani GL, Corbetta M (2007). Electrophysiological signatures of resting state networks in the human brain. Proc Natl Acad Sci USA.

[CR33] Moosmann M, Ritter P, Krastel I, Brink A, Thees S, Blankenburg F, Taskin B (2003). Correlates of alpha rhythm in functional magnetic resonance imaging and near infrared spectroscopy. NeuroImage.

[CR34] Poser BA, Versluis MJ, MHoogduin JM, Norris DG (2006) BOLD contrast sensitivity enhancement and artifact reduction with multiecho EPI: parallel‐acquired inhomogeneity‐desensitized fMRI. Magn Reson Med 55(6):1227–1235. doi: 10.1002/mrm.20900 10.1002/mrm.20900 16680688

[CR35] Scheeringa R, Bastiaansen MC, Petersson KM, Oostenveld R, Norris DG, Hagoort P (2008). Frontal theta EEG activity correlates negatively with the default mode network in resting state. Int J Psychophysiol.

[CR36] Schippers MB, Renken R, Keysers C (2011). The effect of intra- and inter-subject variability of hemodynamic responses on group level Granger causality analyses. NeuroImage.

[CR37] Schölvinck ML, Maier A, Ye FQ, Duyn JH, Leopold DA (2010). Neural basis of global resting-state fMRI activity. Proc Natl Acad Sci USA.

[CR38] Smith SM, Fox PT, Miller KL, Glahn DC, Fox PM, Mackay CE, Filippini N (2009). Correspondence of the brain’s functional architecture during activation and rest. Proc Natl Acad Sci USA.

[CR39] Stephan KE, Weiskopf N, Drysdale PM, Robinson PA, Friston KJ (2007). Comparing hemodynamic models with DCM. NeuroImage.

