# Supplementary figures and images for: Electrophysiological Correlation Patterns of Resting State Networks in Single Subjects: A Combined EEG–fMRI Study
Source: Brain Topogr. 2012 Jun 30;26(1):98–109. doi: 10.1007/s10548-012-0235-0 (PMC3536973; doi:10.1007/s10548-012-0235-0)

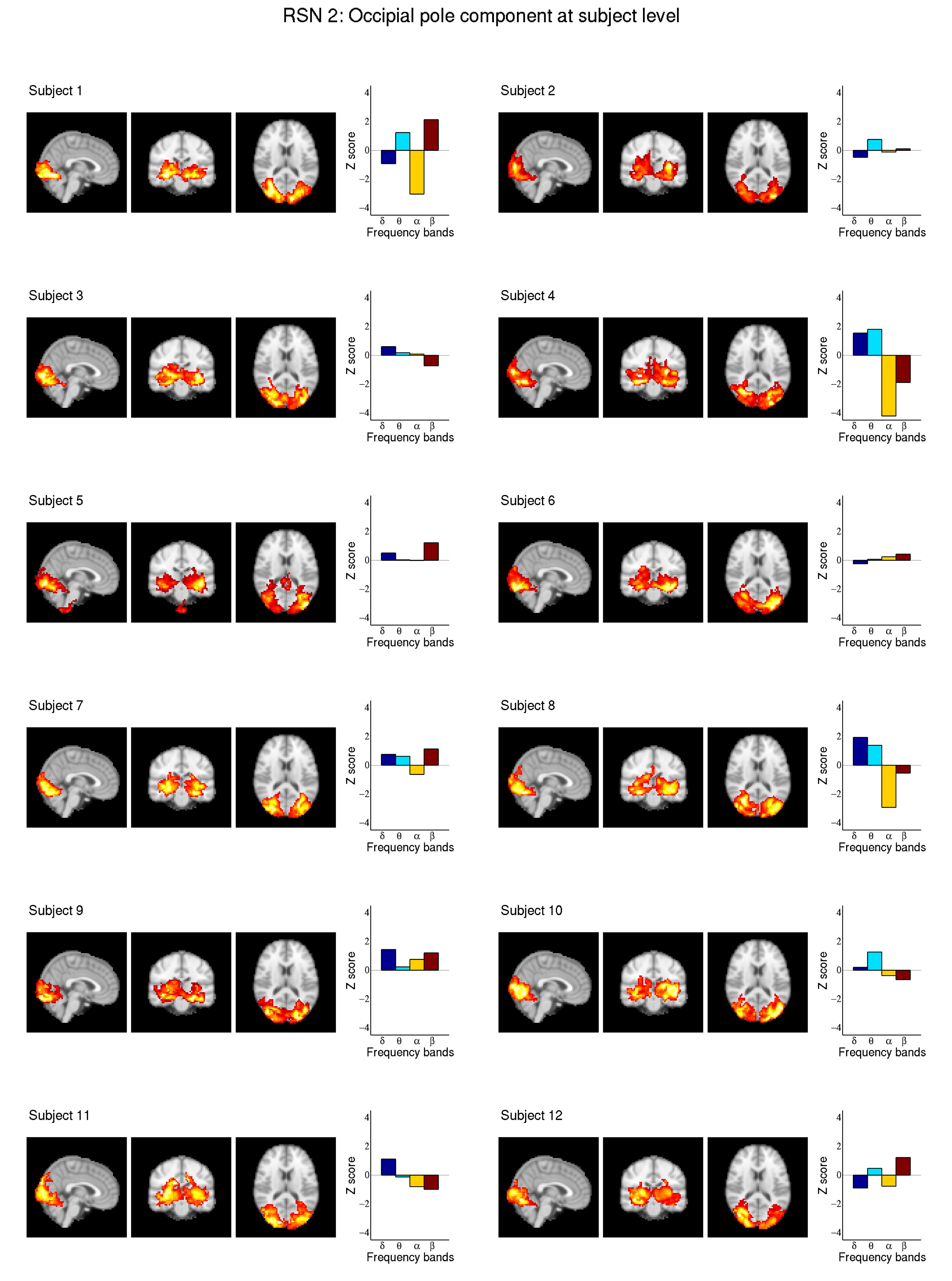

Supplement: Supplementary file 1 — Supplementary material 1 (TIFF 3196 kb) [file 10548_2012_235_MOESM1_ESM.tif]

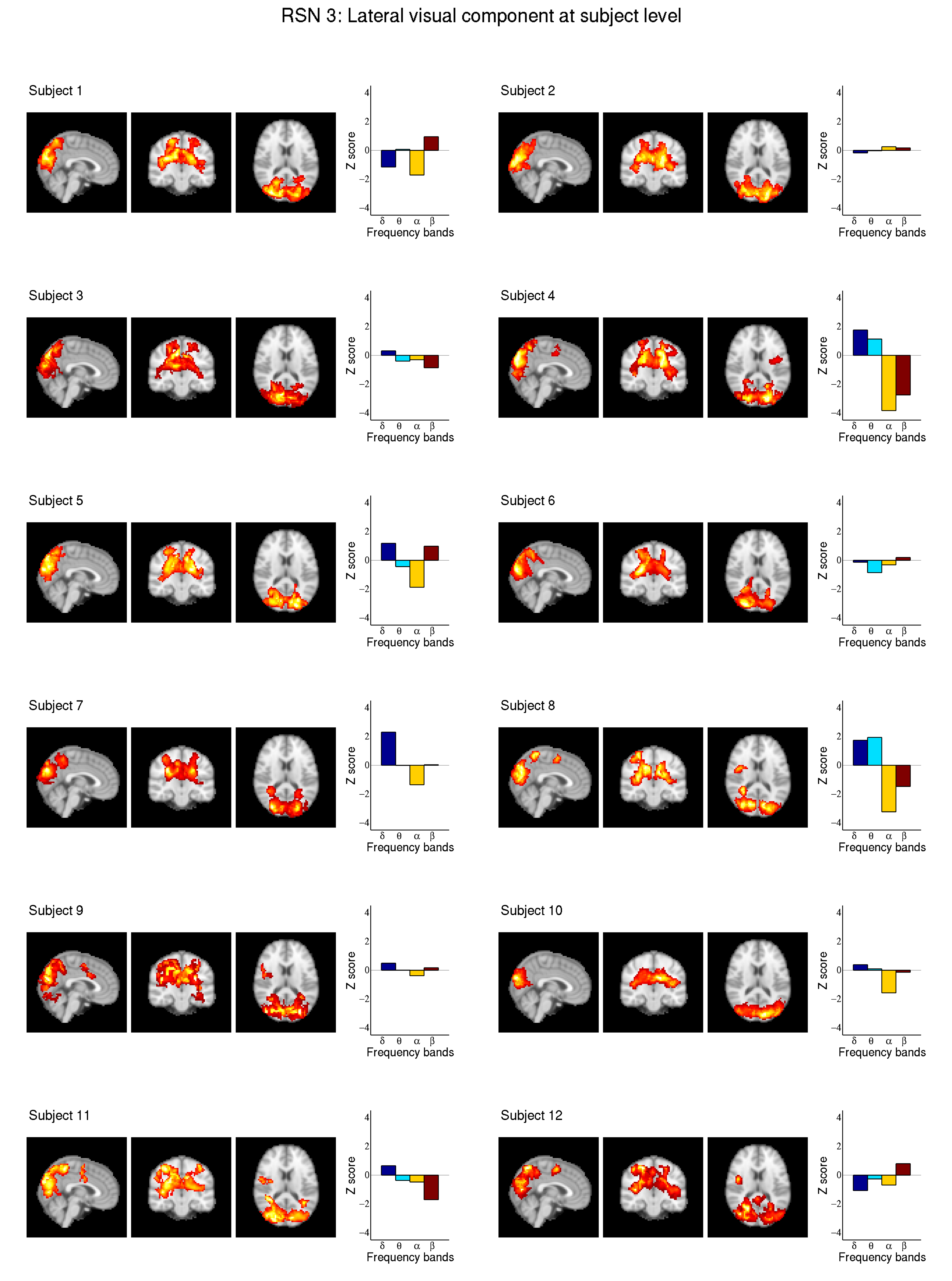

Supplement: Supplementary file 2 — Supplementary material 2 (TIFF 3253 kb) [file 10548_2012_235_MOESM2_ESM.tif]

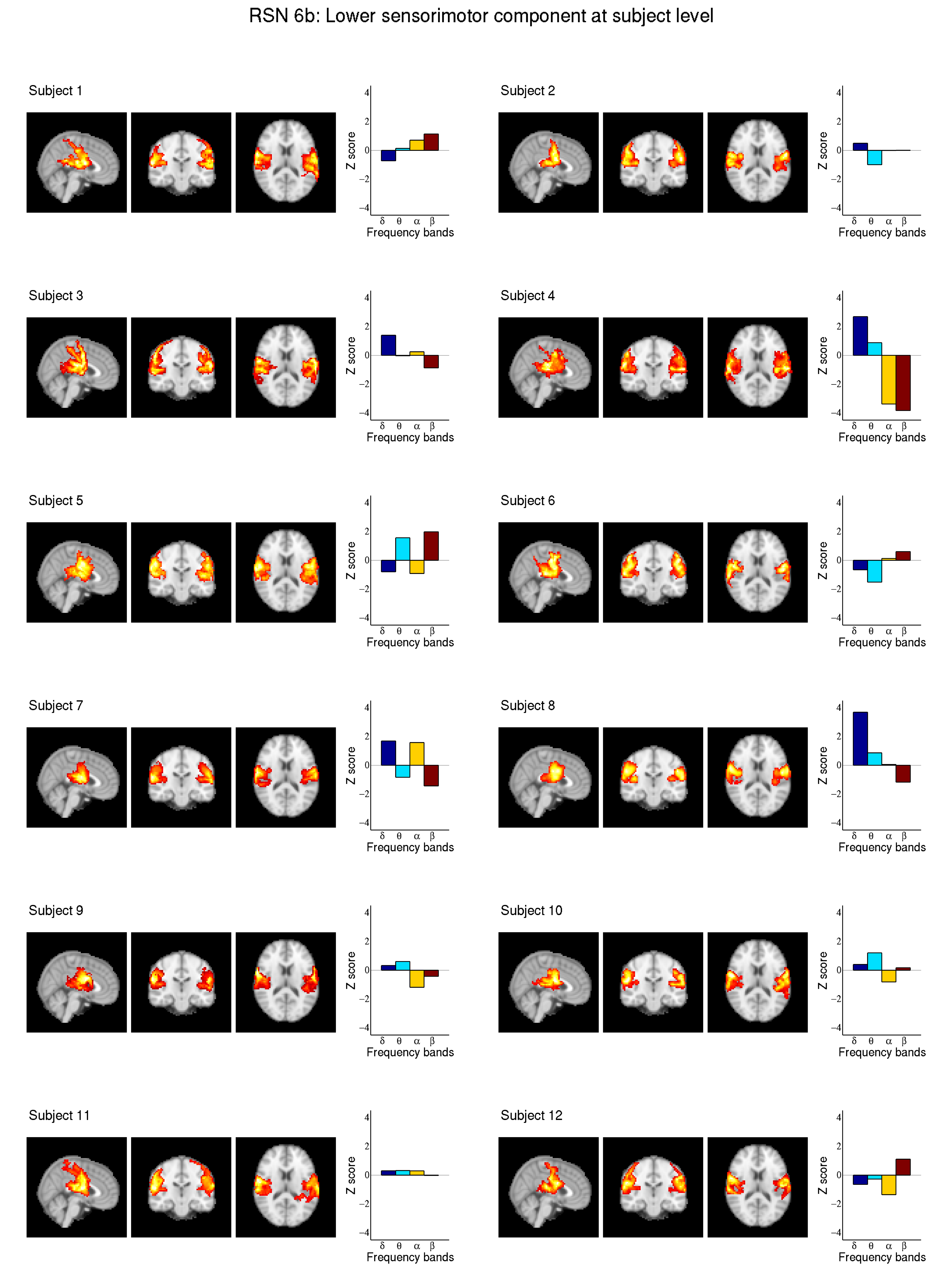

Supplement: Supplementary file 3 — Supplementary material 3 (TIFF 3184 kb) [file 10548_2012_235_MOESM3_ESM.tif]
